# Dual roles of yes-associated protein (YAP) in colorectal cancer

**DOI:** 10.18632/oncotarget.20155

**Published:** 2017-08-11

**Authors:** Chunlin Ou, Zhenqiang Sun, Shen Li, Guiyuan Li, Xiayu Li, Jian Ma

**Affiliations:** ^1^ Hunan Cancer Hospital and the Affiliated Cancer Hospital of Xiangya School of Medicine, Central South University, Changsha, Hunan 410013, China; ^2^ Key Laboratory of Carcinogenesis and Cancer Invasion of the Chinese Ministry of Education, Cancer Research Institute, Central South University, Changsha, Hunan 410078, China; ^3^ Hunan Key Laboratory of Nonresolving Inflammation and Cancer, The Third Xiangya Hospital, Central South University, Changsha, Hunan 410013, China; ^4^ Department of Anorectal Surgery, The First Affiliated Hospital of Zhengzhou University, Zhengzhou, Henan 450052, China; ^5^ Department of Gastrointestinal Surgery, Tumor Hospital of Xinjiang Medical University, Urumqi, Xinjiang 830011, China

**Keywords:** colorectal cancer, Yes-associated protein, tumour biomarker, stemness maintenance, inflammation

## Abstract

Yes-associated protein (YAP) is a downstream effector molecule of a newly emerging tumour suppressor pathway called the Hippo pathway. YAP is a transcriptional co-activator and mis-expressed in various cancers, including colorectal cancer (CRC). Accumulating studies show that the high expression of nuclear YAP is linked with tumour progression and decreased survival. Nuclear YAP can interact with other transcription factors to promote cancer cell proliferation, apoptosis, metastasis and maintenance of stemness. Therefore, YAP has the potential to be a tumour biomarker or therapeutic target for CRC. However, recently, a number of studies have supported a contradictory role for YAP as a tumour suppressor, demonstrating inhibition of the tumorigenesis of CRC, involvement in promoting cell apoptosis, and inhibiting the maintenance of intestinal stem cells and inflammatory activity. In these studies, high expression of YAP was highly correlated with worse survival in CRC. In this review, we will comprehensively summarize and analyse these paradoxical reports, and discuss both the oncogenic and tumour suppressor functions of YAP in the differential status of CRC progression. Further investigation into the mechanisms responsible for the dual function of YAP will be of great value in the prevention, early diagnosis, and therapy of CRC.

## INTRODUCTION

Colorectal cancer (CRC) is the most common malignant tumour of the digestive system and the fourth leading cause of cancer-related death worldwide [1]. According to the Global Cancer Statistic, there were 1.36 million new cases of colorectal cancer, making CRC malignant tumors have the third-highest incidence in the world, ranking third in males and second in females; CRC caused approximately 0.69 million death, ranking fourth among malignant tumors, and increases in mortality rates are still occurring in developed countries [2–5]. However, the five-year survival time of CRC patients is increasing in some populations and varies across countries, ranging from 4.3% to 5.3% for men and from 2.7% to 4.9% for women. Although significant progress has been made in understanding and therapy of CRC, morbidity and mortality rates remain high because cancer recurrence and metastasis are common [6–9]. Therefore, screening and early detection of CRC is an important clinical strategy for improving long-term survival [2, 10]. Currently, clinical screening of CRC commonly involves endoscopic screening, particularly colonoscopy [11–15]; however, there are several problems in this approach, including poor patient compliance, with family history [16, 17], inconvenience, expense and risk [18–20]. Therefore, it is urgent to search for effective strategies for early diagnosis, detection of recurrence, and monitoring of progression in CRC [10].

A tumour biomarker is defined by the National Institutes of Health’s Biomarkers Definitions Working Group as “a characteristic that is objectively measured and evaluated as an indicator of normal biologic processes, pathogenic processes, or pharmacologic responses to a therapeutic intervention” [21]. The expression of tumour biomarkers in cancer cells and tissues can reflect the progression and prognosis of malignant tumour [22]. Yes-Associated Protein (YAP) is a downstream effector molecule of a newly emerging tumour suppressor pathway called Hippo [23, 24]. An increasing number of studies suggest that YAP is an oncogenic transcription coactivator highly expressed in various tumors that can regulate tumour development and progression [25, 26]. However, recently, a number of studies have supported the contradictory view that YAP can be a tumour suppressor because it can function to inhibit the tumorigenesis of CRC through effects on cell growth, apoptosis, maintenance of stemness, and inflammatory responses. In addition, decreased expression of YAP was highly correlated with decreased survival in CRC. This review will comprehensively summarize and analyse these paradoxical reports, and discuss both the oncogenic and tumour suppressor functions of YAP in CRC progression. Further investigation into the mechanisms responsible for the dual function of YAP will be of great value in the prevention, early diagnosis, and therapy of CRC.

### Molecular structures and function of Hippo/YAP

YAP was originally identified in chickens as a binding protein of nonreceptor tyrosine kinase YES1 in 1994 [27], which was considered the mammalian orthologue of *Drosophila* Yorkie (Yki) [28]. The human *YAP* gene maps to chromosome 11 at the 11q22.1 locus. YAP is a proline-rich phosphoprotein containing a proline-rich domain, WW domain, coiled-coil (C-C) domain, and a PDZ-binding motif formed by the four C-terminal amino acids (LTWL) [29–31]. The WW domain is conserved in different YAP protein family members. YAP exists in two isoforms: an isoform with one WW domain is called YAP1 and another isoform with two WW domains is called YAP2 [32, 33]. YAP was originally identified by its association with the YES Src tyrosine kinase and has been shown to be a transcription factor whose cytoplasmic/nuclear shuttling is controlled by post-translational phosphorylation events [34, 35]. As the gene locus of *YAP* is frequently amplified in various human cancers [25, 36, 37], the elevated expression of YAP has been consistently observed in multiple types of human cancers, for example non-small-cell lung cancer (NSCLC) [38, 39], gastric cancer [40, 41], urothelial carcinoma of the bladder (UCB) [42, 43], esophageal squamous cell carcinoma (ESCC) [44, 45], ovarian cancer [46, 47], CRC [48, 49], and cervical cancer [50, 51].

The Hippo pathway is an important signalling pathway in controlling organ size and stem cell self-renewal, and regulates tissue homeostasis, cell proliferation, and apoptosis [37, 52]. Furthermore, recent studies have demonstrated that the Hippo pathway is associated with tumorigenesis and tumour progression and dysfunction of this pathway often contributes to cancer development and tumorigenesis [25, 53, 54]. The components of the mammalian Hippo pathway include manmalian Ste20-like kinase (MST1 and MST2, hippo homologues), large tumour suppressor kinases (LATS1 and LATS2, WTS homologues), Yes-associated protein (YAP), and transcriptional co-activator with PDZ-binding motif (TAZ) [55, 56]. YAP and TAZ have similar structures and functions [26, 57] and are the main effector molecules downstream of the Hippo pathway, which act as transcriptional co-activators. Briefly, when this pathway is activated in mammals, MST1/2 and LATS1/2 kinases cooperate with adaptor proteins containing Salvador 1 (SAV1) and MOB kinase activator 1A/B (MOB1a/b) to phosphorylate and inhibit YAP localized in the nucleus [58–60]. Cytoplasmic YAP is phosphorylated on the Serine127 site (corresponding to mouse S112) by LATS1/2 to bind 14-3-3 site in cytoplasm, subsequently inducing cytoplasmic degradation as a complex of p^S127^-YAP/14-3-3 [61]. However, when the tissue microenvironment changes or cells are stimulated by extracellular or intracellular signals, for example changes in cell polarity, cell-cell contact, mechanical cues, cellular energy status, and ligands of G-protein-coupled receptors(GPCR) [24], the Hippo pathway is inactivated and YAP becomes hyperactivated. As YAP cannot bind DNA directly and must interact with DNA-binding transcription factors, hyperactivated YAP enters the nucleus to bind members of the TEA domain/Transcription Enhancer Factor (TEAD) family [62, 63] or other transcription factors, including RUNX1/2, Smad, ErbB4, and p63/p73, and induces expression of a series of target genes (e.g. *AREG, CTGF, Cyr61*) [23, 64]. Interestingly, Hippo pathway inactivation has been observed in multiple tumors, and elevated expression of YAP has been observed in the nuclei of cancer cells. In this context, YAP can act as a transcriptional co-activator interacting with other transcription factors to regulate cancer cell proliferation, metastasis, stem cell attributes, and patient prognosis.

### Crosstalk of Hippo/YAP with other pathways

**Figure 1:**
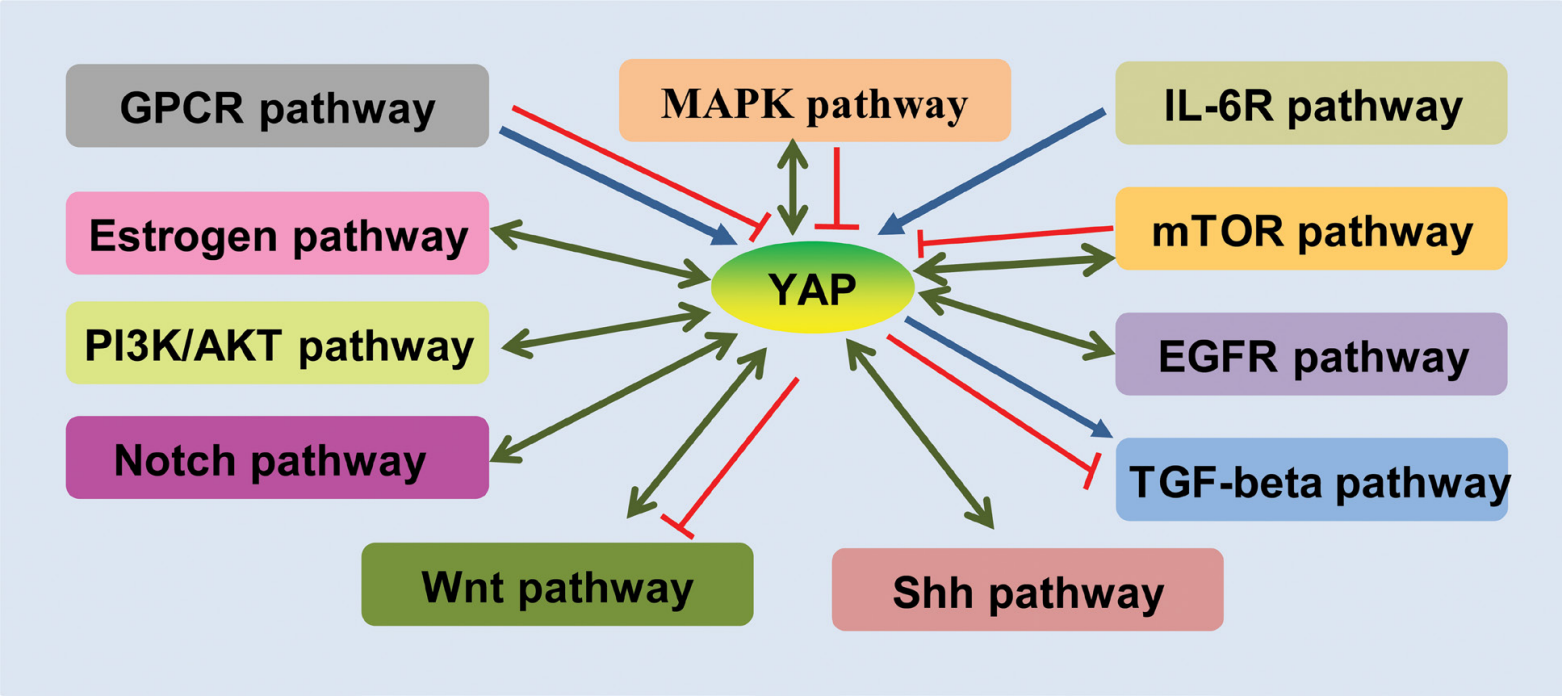
Schematic demonstration of the crosstalks of YAP with other pathways GPCR pathway activates or inhibits the YAP; Estrogen pathway inhibits YAP and YAP activates Estrogen pathway; PI3K/AKT pathway activates YAP and YAP also activates PIK/AKT pathway; Notch pathway activates YAP and YAP also activates PIK/AKT pathway; Shh pathway activates YAP and YAP also activates shh pathway; Wnt pathway activates YAP and YAP activates or inhibits Wnt pathway; YAP activates or inhibits TGF-beta pathway; EGFR pathway activates YAP and YAP also activates EGFR pathway; mTOR pathway activates or inhibits YAP and YAP activates mTOR pathway; IL-6R pathway activates YAP; MAPK pathway activates or inhibits YAP and YAP activates MAPK pathway. In this diagram, the symbol “
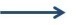
”represents promoted; the symbol “
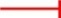
”represents inhibited; and the symbol “
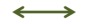
” represents inter-promoted. GPCR, G protein coupled receptors; GPCR, G-protein-coupled receptors; EGFR, epidermal growth factor receptor; Shh, Sonic Hedgehog; PI3K, phosphoinositide 3-kinase.

The progression and development of cancer is a complex process involving in multiple factors and stresses, and is also widely attributed to dysfunction of cellular signalling pathways. YAP is a transcriptional co-activator that is negatively regulated in the Hippo signalling pathway [65, 66]. Recent studies have demonstrated that the Hippo/YAP pathway can crosstalk with other signalling pathways to regulate a series of biological functions in cancer, which is greatly depends on the important role of YAP that is not only mediated by the upstream signal molecule, but can also regulate a series of targets via interacting with transcription factors. By analysing studies published in recent years, we can summarize a complex regulatory network in which YAP is involved in a series of signalling pathways (Figure 1), such as those involving TGF-β/SMAD [67, 68], Wnt/β-catenin [69, 70], epidermal growth factor receptor (EGFR) [39, 71], PI3K-AKT [72, 73], NF-κB [69, 74], Sonic Hedgehog (Shh) [75, 76], mTOR [77, 78], IL6 receptor (IL-6R) [79, 80], GPCR [81, 82], and Notch [83, 84].

Hippo/YAP regulates many biological and pathological features via crosstalk with other signalling pathways, including control of organ size and tissue homeostasis, cell proliferation and apoptosis, and tumorigenesis. Tumaneng et al. [85] demonstrated that YAP inhibits PTEN translation by activating the expression of miR-29, and finally activates the mTOR pathway to regulate organ size. Avruch et al. [86] showed that YAP overexpression promotes colon cancer cell proliferation by synergizing with Wnt/β-catenin signalling. However, Imajo et al. [87] demonstrated that YAP interacts with β-catenin directly and restricts β-catenin nuclear translocation, thereby inhibiting the activation of WNT signaling. The phenomenon could be founded the crosstalk of Hippo/YAP with TGF-β/SMAD signalling pathways. Pefani et al. reported [67] that TGF-β facilitates the YAP/SMAD2 nuclear translocation via targeting Hippo pathway scaffold RASSF1A. Nevertheless, a study by Sun [68] had a contradictory result, demonstrated that YAP inhibits smad3 signaling to promote the survival and self-renewal of tumor initiating cells (TICs) in breast cancer. Lapi et al. [88] reported that p73/YAP directly activated promyelocytic leukaemia (PML) gene transcription during the apoptotic response, and this activity is under the negative control of the proto-oncogenic Akt/PKB kinase. The activation of the Notch signalling pathway is an important pathway for inducing intestinal epithelial regeneration [89]. Camargo et al. [90] revealed that YAP overexpression can activate Notch signalling and c-secretase inhibition, thereby preventing YAP-induced intestinal dysplasia in the intestine; however, a study by Zhou [91] had a contradictory result, demonstrating that the loss of YAP would impair DSS-induced intestinal regeneration by inactivating the Notch pathway. In addition, Fernandez et al. [76] found that YAP functions as a stimulator of cell proliferation and an inhibitor of differentiation possibly downstream of Sonic hedgehog pathway in neural stem cells. Urtasun et al. [92] illustrated that the epidermal growth factor receptor (EGFR) signalling system in human hepatocellular carcinoma (HCC) cells cross-talks with the oncoprotein YAP. Moreover, He et al. [93] demonstrated that YAP can induce the expression of EGFR to control ovarian cancer initiation and progression.

### The dual roles of YAP in CRC cells

### YAP as an oncogene

In mammals, the Hippo/YAP pathway is important in regulating the balance between cell regeneration and proliferation [94]. When the Hippo pathway is dysregulation or inactivated, YAP will be dephosphorylated, thereby translocating into the nucleus to function as a transcriptional co-activator. Nuclear YAP can act as an oncogene that enhances invasion and proliferation and suppresses apoptosis. More than 85% of the YAP protein in normal tissue and cells is found in the cytoplasmic fraction, as determined by immunochemistry (IHC) [95]. However, nuclear YAP overexpression is frequently found in cancer tissues [96–101], including CRC [49, 102, 103]. The molecular mechanism of YAP-mediated CRC regulation is associated with YAP protein phosphorylation and subcellular localization [104]. Konsavage et al. [105] demonstrated that YAP was found in the cytoplasm of the HCT116, SW620, SW480, RKO, LS174T, and HT29 CRC cell lines, and *YAP* gene silencing in SW620 (colon adenocarcinoma) and HCT116 (metastatic CRC) cell lines resulted in reduced growth of colonies in soft agar. Similarly, Wang et al. [106] revealed that YAP expression was highest in HCT116, LS174T, LOVO, SW480 and SW620 in CRC cell lines, and the capacity for proliferation, metastasis, and invasion was dramatically reduced by silencing YAP expression in HCT116 CRC cells. Furthermore, Vigneron et al. [107] showed that cytoplasmic apoptosis-stimulating protein of p53 1(ASPP 1) could inhibit the apoptosis of HCT116 CRC cell lines by suppressing the phosphorylation of YAP and enhancing nuclear accumulation of YAP. In addition, YAP has been identified as driver gene for inducing epithelial–mesenchymal transition (EMT), which may contribute to cancer invasion and metastasis [108–110]. YAP interacts with transcription factor TEAD to induce the multiple downstream targets associated with EMT gene expression [63, 111]. At present, a number of studies have revealed that YAP overexpression is closely related to the EMT of many cancers, such as gastric cancer [112], breast cancer [113], and pancreatic cancer [114]. Some studies have also suggested that YAP is associated with EMT in CRC. Zhao et al. [115] reported that E2A suppresses EMT of CRC cells by inhibiting expression of YAP, which is a downstream target of E2A. In addition, Shao et al. [108] indicated that YAP signalling functionally substitutes for oncogenic KRAS in KRAS-dependent colon cancer cells to regulate the EMT via activation of the transcription factor FOS.

### YAP as a tumour suppressor gene

Although YAP usually functions as an oncogene, abundant literature supports the idea that YAP functions as a tumour suppressor in various cancers, for instance head and neck cancers (HNC) [116], breast cancer [117–119], haematological cancers [120], and CRC [121]. YAP’s function as a tumour suppressor depends on the specific tissues involved [120, 122]. In CRC, YAP has been shown to induce apoptosis in response to DNA damage by enhancing p73 transcription factor function in the promoters of apoptotic genes [34, 88, 118, 123]. Matallanas et al. [117] recently reported that RASSF1A can regulate p73-mediated apoptosis by alleviating YAP cytoplasmic retention, indicating that YAP might play a critical role in tumour suppression. Furthermore, Levy et al. [121] found that in HCT116 CRC cell line, DNA damage downregulates the ubiquitin E3 ligase Itch protein level that can mediate ubiquitination of p73, whereas YAP competes with Itch for binding to p73 to suppress p73 accumulation and induction of apoptosis by cisplatin treatment. Other studies have shown that YAP plays a tumour suppressor role by promoting cell death. Ehsanian et al. [116] demonstrated that overexpression of nuclear YAP causes cell death in HNC cell line. Francesca et al. [120] revealed that low nuclear YAP expression can result in evasion of cell death in the NCI-H929 cell line. Taken together, simply analysing the expression or distribution of YAP cannot be used to determine a therapeutic strategy in CRC, because if YAP can reduce cell proliferation and induce cell apoptosis and death in CRC, it implies that YAP activation could be beneficial in the treatment of CRC.

### The dual roles of YAP in intestinal stem cells

The intestinal epithelium is a monolayer of tightly linked columnar cells [124] with the capacity for rapid self-renewal; it is organized into a crypt-villus unit in which proliferating cells are confined to the crypts, areas of proliferation composed of intestinal stem cells (ISCs) and its daughter-cells [125–127]. Cellular differentiation occurs when the cells move up to the villus tip [128]. ISCs are usually located at the bottoms of crypts and ensure maintenance of the tissue, which can be defined by two essential features, longevity and multipotency [129, 130], and are responsible for this constant self-renewal throughout the lifetime [131]. Studies show that cancer stem cells (CSCs) are closely associated with tumorigenesis and poor prognosis of cancer patients [132, 133]. CSCs are defined as a rare cell population in cancer, and are considered as the origins of tumors, based on histological observations. CSCs display the ability to regulate self-renewal and maintain tumour growth and heterogeneity [130, 134]. Recent studies have revealed that ISCs can act as the cells of origin for intestinal cancer [135, 136], which implies that CSCs originate from ISCs in intestinal crypts. Therefore, further investigation into the mechanisms responsible for the function of ISCs will be of great value to study the pathogenesis of CRC to develop new therapeutic targets.

Recent studies have demonstrated that Hippo/YAP signalling plays an important role in the maintenance of stemness and tissue homeostasis [137–139]. Moreover, it has previously been shown that the YAP protein is primarily localized to the crypt base, and is absent from villi [90, 140], which suggests that YAP may maintain the lack of differentiation of stem cells via binding to the TEAD transcription factor [141]. However, recent studies have shown that YAP has a dual role in regulatory ISCs, which regulate both stem cell proliferation and differentiation. On the one hand, YAP plays a critical role in the maintenance and expansion of undifferentiated ISCs during regeneration. In other words, YAP hyperactivation expands intestinal progenitor/stem cells, while YAP deletion impairs regeneration in intestines damaged with dextran sodium acetate [90, 140]. Camargo et al. [90] showed that ubiquitous overexpression of YAP-S127A in mouse tissues results in loss of differentiation markers and expansion of an undifferentiated cell population in the mouse intestine, whereas Patel et al. [142] showed that YAP can stimulate Notch pathway, which blocks differentiation to suppress the generated ISC tumors. On the other hand, YAP can induce ISC differentiation and restrict ISC expansion during regeneration. Zhou et al. [91] showed that YAP nuclear overexpression promotes the hyperproliferation of ISCs and inhibits differentiation due to activation of Notch signalling. Moreover, YAP can dampen Wnt/β-catenin signalling [143, 144], which is important for the regulation of stem cells [145]; however, YAP nuclear localization is correlated with active Wnt signalling whereas cytoplasmic localization inhibits the Wnt pathway [122]. Barry et al. [122] further showed that YAP restricts the expansion of ISCs as well as critical components of the stem cell niche through suppression of WNT signalling, because YAP interacts with Dishevelled (DVL) [146, 147] to antagonize Wnt signalling and restrict ISC expansion. Taken together, this evidence supports that YAP not only promotes ISC proliferation but also induces ISC differentiation. Together, these studies provide strong evidence that YAP1 functions as a stem cell regulator and imply that YAP plays important roles in maintaining stemness and tissue homeostasis.

### The dual roles of YAP in CRC-associated inflammation response

Cancer has been considered as a process of tissue repair dysregulation as well as being called “Wounds That Never Heal” [148]. Mantovani [149] reported that inflammation continually accelerates the “inflammation-cancer chain” in tumorigenesis, which may eventually evolve into “nonresolving inflammation-related cancer.” To some degree, the relationship between cancer and inflammation has been confirmed. Epidemiologic evidence suggests that approximately 25% of all human cancer worldwide may be caused by inflammation [150, 151]. In addition, inflammatory cells that can infiltrate tissue are always found in the cancer microenvironment. In tumorigenesis, inflammatory cells and molecules influence almost every aspect of cancer, including the formation of the cancer microenvironment [152], invasion/metastasis [153], and immune escape [154–156]. Consequently, biologically malignant inflammation is regarded as “the seventh characteristic of tumors” [149, 157–159].

Recent studies have demonstrated that inflammation plays a key role in the development and progression of CRC [160–162]. YAP plays a vital role in inflammation-induced cancer because it can act as a transcriptional co-activator interacting with other transcription factors to modulate expression of inflammation-associated factors. However, YAP not only induced inflammation, but also reduced inflammation according to the function of inflammation-associated factors. Yamada et al. [163] and Li et al. [164] showed that the biliary mitogen IL-33 facilitates oncogene-induced cholangiocarcinoma (CCA) in mice through constitutively activating AKT and YAP oncogenes. Taniguchi et al. [80] have shown that IL-6 family members can be activated upon receptor engagement to phosphorylate YAP and induce its stabilization and nuclear translocation in CRC cell lines, promoting inflammatory bowel diseases (IBD) and CRC. NF-κB is an important mediator of inflammation [165], pathogenesis of intestinal inflammation, and inflammatory bowel disease (IBD) [166]. There is a negative correlation between YAP and NF-κB signalling pathway. For instance, Gao et al. [167] demonstrated that knockdown of the expression of *YAP* by shRNA interference increases the luciferase activities of AP-1 and NF-κB in 293T cells, inducing expression of various target genes related to proliferation, angiogenesis, apoptosis and inflammation. In addition, Gordon et al. [74] reported that the tyrosine phosphorylation of YAP can induce the expression of pro-apoptotic genes to drive intestinal epithelial apoptosis. On the other hand, the tyrosine phosphorylation of YAP can restrict NF-κB-dependent inflammation to promote recovery from inflammation-induced injury and maintain epithelial homeostasis. Furthermore, Kim et al. [48] showed that the activated YAP1 not only promoted colon regeneration after colitis, but also induced the proliferation of colon cancer cell lines in mice. Similarly, Huang et al. [168] demonstrated that the deletion of YAP increased astrocytic activation in culture and *in vivo* by hyperactivating the JAK-STAT inflammatory pathway, and negatively controlled neuroinflammation through the YAP-SOCS pathway.

### Clinical relevance in CRC

### Expression of YAP as prognostic factors in CRC

Overexpression of nuclear YAP has been observed in multiple types of human cancers [38–51] and is significantly associated with worse overall survival [46, 169–171]. In our previous study, Sun et al. [172] showed through meta-analysis of 21 studies that YAP overexpression was closely associated with adverse effects on overall survival (OS) and disease free survival (DFS) in numerous cancers. As mentioned, the expression of YAP in most types of cancers is higher than that in the corresponding normal tissues or cells. Studies have shown that the expression of YAP is highly correlated with the pTNM stage, nodal status, and tumour status of CRC, and that nuclear YAP overexpression was closely associated with worse overall survival [105, 106, 173–175]. Wang et al. [175] demonstrated that nuclear YAP overexpression was observed in approximately 52.5 % of 139 CRC cases. Similarly, Konsavage et al. [105] reported that 86% of 36 primary CRC tumors scored positively for nuclear localization of YAP. Consistent with a study by Wang et al. [106], our previous study [102] showed that high expression of YAP is closely associated with lymph node metastasis and strongly linked to worse overall survival in CRC.

However, other reports demonstrating losses of expression of nuclear YAP in various cancers, including HNSCC [116], breast cancer [118, 119], haematological cancers [120], and CRC [121], demonstrate that contradictory reports exist on the expression of YAP in cancers including CRC. Barry et al. [122] showed that loss of YAP expression was associated with high grade tumors and stage IV cancer by evaluating YAP expression in a cohort of 672 CRC samples using immunohistochemistry, which may imply that YAP may act as a tumour suppressor in human CRC. Similarly in breast cancer, Kim et al. reported that stromal YAP and pYAP expression in breast cancer was associated with shorter DFS and OS [176]. Taken together, it is unclear how YAP can serve as a prognostic marker for CRC progression. Therefore, it is necessary to clarify the relationship between the expression of YAP and its clinical implications in CRC, which will aid in a better understanding the functions and roles of YAP in the development and progression of CRC.

### The therapeutic strategies for YAP in CRC

Despite tremendous progress in CRC therapy, the morbidity and mortality caused by CRC is still high worldwide. Encouragingly, numerous studies showed that therapeutic efficacy for CRC patients could be improved by screening and early detection of CRC followed by timely intervention with surgery, chemoradiotherapy and targeted therapy. However, it was noted that the ideal tumour marker—described as being able to identify patients with an extended risk of fast progression or early recurrence after operation when compared with other patients of the same age, disease stage and other characteristics—is still lacking in CRC therapy. Endoscopic screening carries a certain degree of risk [16–20]. Therefore, it is urgent to search for the ideal molecular markers and drug targets for CRC.

**Figure 2:**
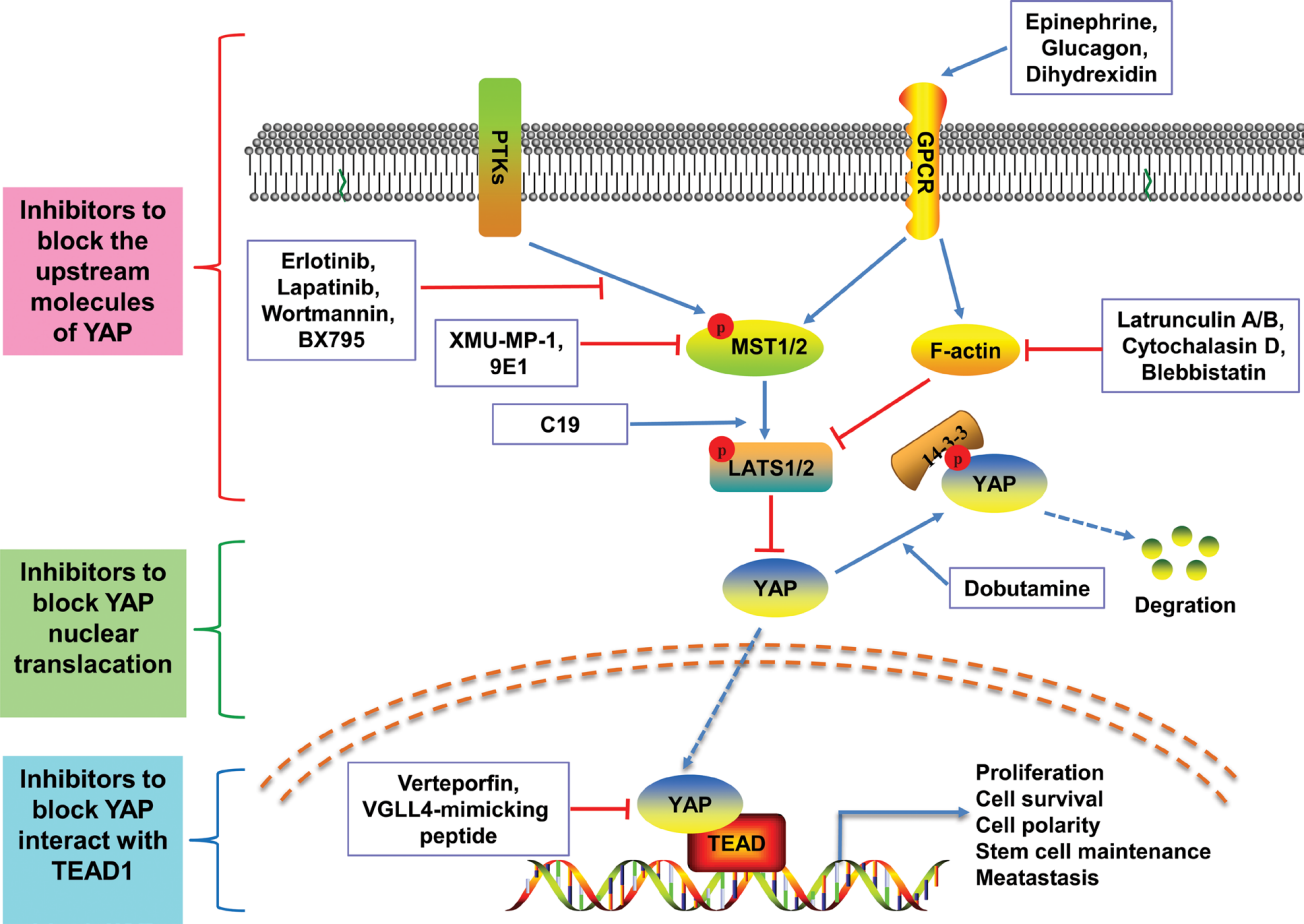
Schematic demonstration of the small-molecule modulators of YAP These small-molecule modulators of YAP can be classified into three categories: (1) the red area represents those regulating the upstream molecules of YAP, as follows: C19 inhibits the activated of LATS1; XMU-MP-1 and 9E1 targets MST1/2; Latrunculin A/B, cytochalasin D and Blebbistatin directly inhibit F-actin; the GPCR agonists (e.g. Epinephrine, Glucagon, Dihydrexidine) activate LATS1/2; Erlotinib, Lapatinib, Wortmannin and BX795 inhibit the activated of MST1/2; (2) the green area represents those modulating the phosphorylation of YAP and blocking YAP nuclear translacation, such as Dobutamine; (3) the blue area represents those inhibiting YAP interaction with TEAD1 by directly targeting YAP, such as Verteporfin, VGLL4-mimicking peptide.

Small-molecule therapeutics are currently the main strategy for personalized treatment of advanced cancer [104]. Because the Hippo pathway is evolutionarily conserved, the core molecules of the Hippo pathway are rarely mutated. YAP may be a potential target for small-molecule modulators. These small-molecule modulators of YAP can be classified into three categories (Figure 2): (1) those regulating the upstream molecules of YAP, thereby effecting YAP–TEAD transcriptional activity, such as C19 [177], XMU-MP-1 [178], 9E1 [179], Latrunculin A/B [180], cytochalasin D [181], Blebbistatin [182], Erlotinib [183], Lapatinib [184], Wortmannin [71], BX795 [71] and the GPCR agonists(e.g. Epinephrine, Glucagon, Dihydrexidine [180]); (2) those modulating the phosphorylation of YAP and blocking YAP nuclear translacation, such as Dobutamine [185]; (3) those directly inhibiting YAP interact with TEAD1, directly targeting YAP, such as Verteporfin [186], VGLL4-mimicking peptide [187]. Fortunately, therapies targeting YAP have been breakthroughs in many cancers. For example, Brodowska et al. reported that the photosensitizer Verteporfin (VP) can inhibit YAP–TEAD transcriptional activity, thereby suppressing retinoblastoma(Rb) cell growth [186]. Similarily, Jiao et al. [187] demonstrated that a peptide mimicking VGLL4 functions as a physical antagonist of YAP and blocks YAP oncogenic activity at the transcriptional level in gastric cancer, which may provide an option for therapy. However, there have been few reports of small-molecule modulators of YAP being applied in treatment of CRC. Recent studies examining YAP in CRC have suggested that YAP may act synergistically with chemotherapy drugs. Lee et al. [174] demonstrated that the group of patients with activated YAP in CRC (AYCC) had slightly more advanced disease and much shorter survival rates than another group of patients with inactivated YAP1 in CRC. YAP activation was significantly associated with poor response to cetuximab therapy. Furthermore, Touil et al. [188] found that YAP could be a potential molecular target in dormant micrometastases during 5FU chemotherapy in colon cancer cells. Similarly, Huang et al. [48] showed that the treatment of ovatodiolide in combination with 5-FU significantly suppressed YAP1 oncogenic pathways to inhibit M2 TAM generation and the tumorigenesis of CRC. With regards to other types of cancers, Wang et al. [189] reported that simvastatin induced cancer cell growth arrest and decreased nuclear YAP by interfering with protein geranylgeranylation in breast cancer, and [190] illustrated that the activation of YAP can increase cell proliferation and methotrexate/doxorubicin resistance in osteosarcoma cells. We speculate that the reason for limited clinical application of small-molecule modulators of YAP is because it is difficult to determine whether inhibiting or stimulating the expression of YAP would be a more suitable strategy, due to both the oncogenic and tumour suppressor roles of YAP observed in CRC. Therefore, the role of YAP should be further explored.

## CONCLUSIONS AND FUTURE PERSPECTIVES

In the last several years, the rapid progress of Hippo/YAP pathway research has resulted in a broad signalling map being built, and many studies have demonstrated that the effector molecule YAP is closely associated with the physiological organ size control and pathological progression of CRC. Tumour biomarkers have attracted increasing attention as novel tools in cancer diagnosis and therapy. Currently, it is a boom time for therapies targeting Hippo/YAP in CRC. In many studies, YAP has displayed the characteristics of a tumour biomarker because it is overexpressed in many cancers, promotes the development and progression of cancers, is easily detected in tissue, and is associated with prognosis. However, a number of studies have suggested that YAP plays a role as a tumour suppressor in the development and progression of CRC. By comprehensively analysing relevant research, we speculate that this paradox can be explained in four ways: (1) As an effector molecule of the Hippo signalling pathway, YAP can act as a bridge in crosstalk with other signalling pathways(e.g. TGF-β/SMAD, Wnt/β-catenin), and can modulate the activity level of these pathways via positive or negative regulation, depending on the type of tissues or cells; (2) YAP activity is regulated by the upstream tumour suppressor signalling of the Hippo pathway; on the other hand, YAP can transcriptionally modulate the upstream tumour suppressor molecules of the Hippo pathway (e.g. Lats2 [191], NF2 [192]) to inhibit CRC tumorigenesis; (3) In the different stages of CRC development and progression, the distribution of nuclear vs. cytoplasmic YAP expression and the phosphorylation level of cytoplasmic YAP change dynamically; (4) In poorly differentiated CRC tissue, although the expression of YAP is mainly localized to the nucleus, the transcriptional activity of YAP is suppressed because other nuclear molecules (e.g. T-cell lymphoma invasion and metastasis 1 (TIAM1) [193], IQ motif containing GTPase activating protein 1 (IQGAP1) [194]) bind to YAP and occupy its TEAD-binding domain; thus, YAP is unable to bind the transcription factor TEAD1 and regulate target gene expression [195]. The development and progression of CRC is a dynamic process, and the expression levels of some molecules differs in the different stages of CRC. This implies that YAP may change from a tumour inducer to a tumour suppressor under some conditions. Important goals for ongoing tumour biomarker studies include ensuring that patients receive the benefit of being exposed to as many active therapies as possible while minimizing any treatment-related morbidity. Therefore, greater understanding of the clinical roles and molecular mechanisms of YAP in CRC is required and will be of great value in the development of new molecular targets for drugs. With all these efforts, targeting YAP may become a promising therapeutic strategy for the treatment of CRC in the future.

## Abbreviations

YAP: Yes-associated protein
CRC: colorectal cancer
CEA: carcinoembryonic antigen
CA19-9: carbohydrate antigen 19-9
NSCLC: non-small-cell lung cancer
UCB: urothelial carcinoma of the bladder
ESCC: esophageal squamous cell carcinoma
Yki: Yorkie
MOB1a/b: MOB kinase activator 1A/B
SAV1: Salvador 1
LATS1: large tumour suppressor kinases 1
LATS2: large tumour suppressor kinases 2
MST1: manmalian Ste20-like kinase 1
MST2: manmalian Ste20-like kinase 2
TEAD: TEA domain/Transcription Enhancer Factor
GPCR: G-protein-coupled receptors
EGFR: epidermal growth factor receptor
Shh: Sonic Hedgehog
PI3K: phosphoinositide 3-kinase
PDK1: phosphoinositide-dependent kinase
PML: promyelocytic leukaemia
HCC: hepatocellular carcinoma
IHC: immunochemistry
ASPP 1: apoptosis-stimulating protein of p53 1
EMT: epithelial–mesenchymal transition
HNC: head and neck cancers
ISCs: intestinal stem cells
CSCs: cancer stem cells
DVL: Dishevelled
CCA: cholangiocarcinoma
IBD: inflammatory bowel diseases
DFS: disease free survival
OS: overall survival
TIAM1: T-cell lymphoma invasion and metastasis 1
IQGAP1: IQ motif containing GTPase activating protein 1
